# Computational analysis suggests that virulence of *Chromobacterium violaceum* might be linked to biofilm formation and poly-NAG biosynthesis

**DOI:** 10.1590/S1415-47572009000300031

**Published:** 2009-09-01

**Authors:** Sidnei Becker, Cíntia Soares, Luismar Marques Porto

**Affiliations:** Laboratório de Tecnologias Integradas, Departamento de Engenharia Química e de Alimentos, Universidade Federal de Santa Catarina, Florianópolis, SCBrazil

**Keywords:** biofilms, exopolysaccharide, *Chromobacterium violaceum pathogenicity*, comparative genomics

## Abstract

Groups of genes that produce exopolysaccharide with a *N*-acetyl-D-glucosamine monomer are in the genome of several pathogenic bacteria. *Chromobacterium violaceum*, an opportunistic pathogen, has the operon hmsHFR-CV2940, whose proteins can synthesize such polysaccharide. In this work, multiple alignments among proteins from bacteria that synthesize such polysaccharide were used to verify the existence of amino acids that might be critical for pathogen activity. Three-dimensional models were generated for spatial visualization of these amino acid residues. The analysis carried out showed that the protein HmsR preserves the amino acids D135, D228, Q264 and R267, considered critical for the formation of biofilms and, furthermore, that these amino acids are close to each other. The protein HmsF of *C. violaceum* preserves the residues D86, D87, H156 and W115. It was also shown that these residues are also close to each other in their spatial arrangement. For the proteins HmsH and CV2940 there is evidence of conservation of the residues R104 and W94, respectively. Conservation and favorable spatial location of those critical amino acids that constitute the proteins of the operon indicates that they preserve the same enzymatic function in biofilm synthesis. This is an indicator that the operon hmsHFR-CV2940 is a possible target in *C. violaceum* pathogenicity.

## Introduction

Some microorganisms develop cooperative strategies in the formation of biofilms. Biofilm can be defined as interdependent communities of microorganisms, usually connected with a surface presenting high resistance to environmental stress. It consists of a complex symbiotic system of great importance to biotechnological and medical applications, since it is correlated with bacterial resistance to antibiotics and promotion of lethal infections.

The formation of biofilms in several microorganisms involves the presence of a complex matrix where the polymer poly(beta-1,6-*N*-acetyl-D-glucosamine), poly-NAG, can play an important role (Itoh *et al.*, 2005), possibly related to resistance to antibiotics and promotion of bacterial infections. Poly-NAG has been implicated in the formation of biofilms in several pathogen bacteria. The polymer, which is involved in cell adhesion in *Staphylococcus epidermidis* (Mack *et al.*, 1996), is also involved in abiotic surface binding, and in intercellular adhesion formation of biofilm in *Escherichia coli* (Itoh *et al.*, 2005).

The *hms*HFRS operon has been involved in the formation of biofilms *in vitro* in *Yersinia pestis*. This operon was related to the blockage of the digestive system of fleas, and it is associated with the transmission of *Y. pestis* to mammals (Jarrett *et al.*, 2004). The bacterium *Bordetella pertussis* has the operon *bps*ABCD related to a polymer that contributes to the formation of biofilms in the respiratory tract of rats (Sloan *et al.*, 2007). An understanding of the relationship between the operons of those bacteria in the formation of biofilms could potentially lead to the development of a vaccine applicable to different bacterial species.

Recently, the sequencing of the complete genome of the *Chromobacterium violaceum*, strain ATCC 12472 (Brazilian National Genome Project Consortium, 2003; www.brgene.lncc.br; www.ncbi.nlm.nih.gov: access number NC_005085) was carried out to promote domestic genome projects. The investment was justified due to the great biotechnological and pharmaceutical potential of this microorganism. *Chromobacterium violaceum* is considered a possible pathogen for humans and animals, with several reported cases of infection in humans and animals in tropical and subtropical areas, where it is normally found. Its potential pathogenicity in humans was first described in 1927 in Malaysia (Sneath *et al.*, 1953). Although cases of infection with *C. violaceum* are rare, it has a mortality rate of more than 57%, as reported in a case of chronic granulomatous disease in children (Macher *et al.*, 1982). *C. violaceum* reveals some potentially pathogenic genes (Brazilian National Genome Project Consortium, 2003); however, their extent is not yet known or how these genes are organized in their genome.

This work aims to link, through comparative analysis, the genes *hms*H, *hms*F, *hms*R and the ORF CV2940 of *C. violaceum* with the genes of organisms known and related to the formation of biofilm in an effort to shed light on the pathogenic potential of *C. violaceum*. The analysis of the *C. violaceum* genome reveals the presence of possible genes involved in the formation of biofilms, and the sequence of the group of genes *hms*HFR and CV2940 are potentially functional for it. Initially, the relationship among the proteins is carried out by the alignment of amino acid sequences and it is complemented by structural modeling in order to identify the importance of the amino acid through spatial viewing.

## Methods

Comparisons were carried out using the amino acid sequences of the organisms listed in Table 1 and obtained from GenBank. These organisms with their genomic regions were selected for this study because of their confirmed connection with biofilm formation or production of polysaccharides such as poly(beta-1,6-*N*-acetyl-D-glucosamine).

An initial approach among groups of proteins (Table 1) was performed using the Clustal W server (Jeanmougin *et al.*, 1998) through multiple alignments in order to identify possible amino acids conserved among the proteins. The structural modeling was performed by 3D-JIGSAW (Bates *et al.*, 2001) and PHYRE servers (Kelley *et al.*, 2000) with the models with higher identity chosen as the template. The ProCheck program (Laskowski *et al.*, 1996) was used to check the quality of the three-dimensional model generated. The quality of the model was analyzed through the Ramachandran diagram (Ramachandran *et al.*, 1963). Analyses were carried out using the Verify3D program (Eisenberg *et al.*, 1997) in order to ensure a more solid and comprehensive quality of the model.

## Results

**Figure 1:**
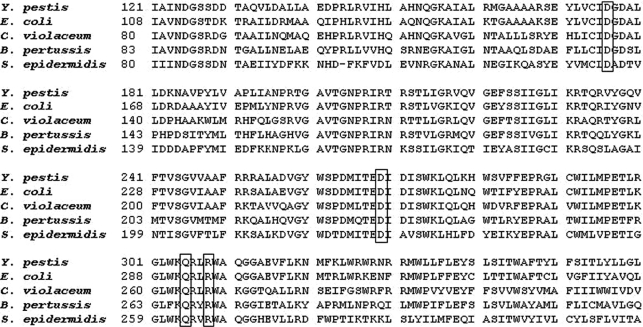
Partial multiple alignment of HmsR from *C. violaceum* compared with HmsR of *Y. pestis*; PgaC of *E. coli*; IcaA of *S. epidermidis* and HmsR of *B. pertussis*. The critical residues aspartate (D), glutamine (Q) and arginine (R) are demarcated.

In a multiple alignment comparison generated by the Clustal W server with other glycosyltransferase proteins, the critical amino acids related to formation of biofilms are preserved also in the HmsR protein of *C. violaceum* (Figure 1). Conserved amino acids that are critical for function of HmsR of *Y. pestis* (D176, D269, Q305 and R308) correspond to residues D135, D228, Q264 and R267 in *C. violaceum,* respectively.

**Figure 2:**
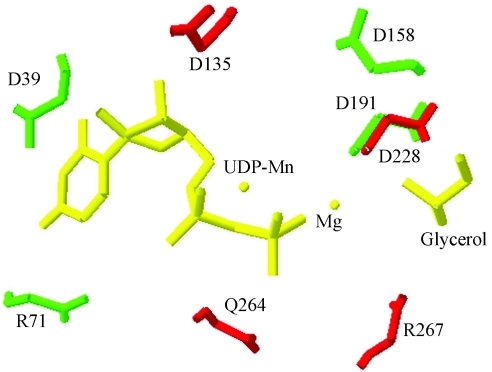
Superimposed proteins closing up the active site of SpsA protein bound to Mn-UDP with the built model of HmsR protein *C. violaceum*. The residues of SpsA are green with HmsR residues in red and the UDP-Mn, Mg and Glycerol in yellow.

The structural model generated (Figure 2) comprises the critical amino acids for the function of this protein in the formation of biofilms arranged in a three dimensional conformation with the possibility of fitting a substrate. The residue D228 is in exactly the same position as D191 (SpsA) with other conserved residues (D135, Q264 and R267) also positioned on the active site.

**Figure 3:**
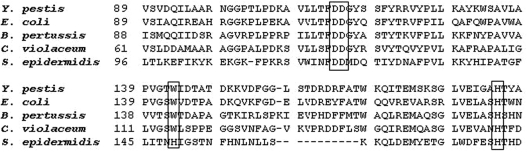
Multiple partial alignment of HmsF of *C. violaceum* compared with PgaB of *E. coli;* IcaB of *S. epidermidis*; HmsF of *Y. pestis* and HmsF of *B. pertussis*. The critical residue to extracellular matrix formation in *Y. pestis*: D (aspartate), W (tryptophan) and H (histidine) are demarcated.

The multiple alignment among organisms in this study shows that the polypeptide HmsF of *C. violaceum* retains the amino acids that are crucial to the formation of extracellular matrix in biofilms (Figure 3). The D114 and D115 aspartate amino acids of the protein HmsF of *Y. pestis*, which have HmsF deacetylase activity functionality, are conserved on all related organisms in this study, and in *C. violaceum* correspond to aspartate D86 and D87. The important amino acids in the synthesis of extracellular matrix in *Y. pestis* (W143 and H184), which correspond in *C. violaceum* to W115 and H156, are also retained.

**Figure 4:**
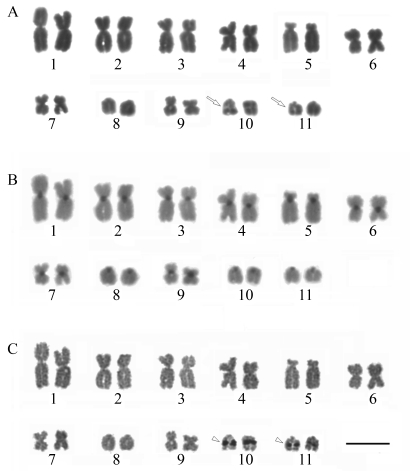
Superimposed proteins *Sp*PgdA and HmsF. The residues of *Sp*PdgA are in green, the HmsF residues are in red and Zinc is in black.

In the three-dimensional model of HmsF of *C. violaceum* obtained (Figure 4), the 3D-JIGSAW server made use of the protein *Sp*PgdA as a template (PDB code: 2c1g) (Blair *et al.*, 2005), which is a *N*-acetyl-glucosamine deacetylase. The model generated for HmsF of *C. violaceum* presenting the spatial location of critical residues in the biofilm formation to *Y. pestis* shows that the residues D86, D87, W115 and H156 are closely located in space (Figure 4). These residues are situated around the active site.

In the multiple alignment of HmsH protein, it was observed that the residue arginine R113, which had a poor performance in the formation of biofilms in *Y. pestis* (Forman *et al.*, 2006), and that in *C. violaceum* is the residue R104, was conserved in almost all organisms studied, but it was not observed in *S. epidermidis*, a Gram-positive bacteria (not shown). The multiple alignment performed among bacteria of this study for ORF CV2940 shows that the critical residue tryptophan W80 of *Y. pestis* is also conserved in Gram-negative bacteria *E. coli* and *C. violaceum*, corresponding to the residue W94 in *C. violaceum*.

The analysis of the three-dimensional model generated for HmsR of *C. violaceum* by the Ramachandran diagram shows that the model holds 99.1% of the residues in allowed regions, demonstrating a good quality. The analysis of the environment of each amino acid residue carried out by the Verify3D program presented two regions (residues 175 to 187 and 230 to 237) in the HmsR with a negative 3D-1D score, indicating a probable incorrect folding in this region. However, the critical residues for HmsR in the formation of biofilm do not appear in this region. The structural model of HmsF protein generated also presents a good quality, with a Ramachandran plot showing 98.5% of the residues in allowed regions. The model also shows a good quality for HmsF when compared with the results obtained in the Verify3D program, providing a great number of amino acids in a positive 3D-1D score.

## Discussion

The production of biofilms by several organisms suggests the conservation of proteins or a set of proteins with similar function for its synthesis.

The HmsR protein, an important glycosyltransferase in polysaccharide synthesis, maintains the critical residues for its function also in *C. violaceum*. Critical residues in the formation of biofilms in HmsR protein of *Y. pestis* (Forman *et al.*, 2006), which match in *C. violaceum* to aspartate D135 and D228, arginine R267 and glutamine Q264, are retained in all the bacteria shown in this study, indicating functional similarity for this protein. The spatial location of the critical residues for biofilm formation in an active site strongly emphasizes the enzymatic role that the HmsR protein of *C. violaceum* can perform in the synthesis of polysaccharide.

The HmsF protein of *C. violaceum* retains all the amino acids that are critical to the formation of biofilms. The generated model with the spatial location of critical residues in the formation of biofilms in *Y. pestis* shows that they are close to each other. The use of a deacetylase as template was important due to the role that this enzyme plays in the formation of biofilms. The deacetylation involves mainly aspartate (D) and histidine (H) residues (Blair *et al.*, 2005), and in this work these residues are located in a favorable spatial condition. Although actual evidence can be obtained from additional techniques such as substrate-enzyme reactions, it can be assumed that this model has a deacetylase catalytic region. The protein HmsH preserves the residue arginine, which shows a moderate effect on the formation of biofilms in *Y. pestis* among all bacteria of this study, except for *S. epidermidis* which is a Gram-positive bacterium. In the three-dimensional model, the choice of the template of the OGT enzyme (PDB accession code 1w3b) was very useful, since this enzyme has an *N*-acetylglucosamine (GlcNac) additive activity (Jinek *et al.*, 2004) that is the basic unit of the polymer poly(beta-1,6-*N*-acetyl-D-glucosamine).

Conservation and favorable spatial arrangement of those critical amino acids that constitute the proteins encoded by the elucidated operon indicate that they may express the necessary enzymatic function for resistant biofilm formation. Therefore the conclusion is that the operon *hms*HFR-CV2940 might be linked to *C. violaceum* pathogenicity.

## Figures and Tables

**Table 1:** Bacteria with their genomic regions used in the comparative study.

Organism and GenBank access number	GenBank access number	Gene/ORF
*Chromobacterium violaceum* ATCC 12472 NC_005085	NP_902610 NP_902611 NP_902612 NP_902613	CV2940 *hms*R *hms*F *hms*H
*Escherichia coli *K12 NC_000913	P69435 P75906 P75905 P69433	*pga*A *pga*B *pga*C *pga*D
*Staphylococcus epidermidis *DQ149646	AAZ78357 AAZ78358 AAZ78359 AAZ78360	*ica*A *ica*D *ica*B *ica*C
*Yersinia pestis *KIM6+ YPU22837	AAB66588 AAB66589 AAB66590 AAB66591	*hms*H *hms*F *hms*R *hms*S
*Bordetella pertussis *Tohama I NC_002929	NP_880625 NP-_880626 NP_880627 NP_880628	BP1941 *hms*R* hms*F* hms*H
